# Everybody's Page

**Published:** 1893-01-21

**Authors:** 


### EVERYBODY'S PAGE.

A CLERICAL correspondent points out that boycotting has many sympathizers who profess a belief in the Bible. Their attention may well be called to Rev. xiii. 17, a passage in which a kingdom or state of society is represented under the reign of the Beast, the wearing of whose mark, name, or number is the only condition upon which buying or selling is permitted. All persons who decline this condition are boycotted.

---

SOME indignation has been felt amongst Americans lately at the very unpromising specimens of the medical profession put down to their nationality. Neill, they protest, originally hailing from Glasgow, is termed the "American" Poisoner, whilst two miscreants, natives of Belfast, and adopting the titles of "The Faculty of American Physicians and Surgeons," are described in the English press as "the American doctors." This is a little hard, but it is flattering also, for it suggests the idea that the "American stamp" is one which is so respected as to be a species of certificate without which these adventurers could not hope for such success.

---

A FEMALE lunatic has been figuring in a somewhat new light at a Shropshire workhouse, a sad tragedy, consequent on her presence, or rather, on her absence, disclosing the fact that this woman was actually given the responsible task of taking care of the children inmates of the establishment. It appears that during a short absence of their "caretaker," the small paupers were left playing together, when one child, taking advantage of the unexpected leisure hour, took a red-hot poker from the neighbouring fire, and ignited the clothes of his companion. The little boy succumbed to the injuries last week. The jury, after inquiring into the facts of the case, suggested an improvement in the management of the children, whereby a woman, not a registered lunatic, should be appointed their caretaker.

---

THE answer which the Archbishop of Canterbury has given to the question lately addressed him, why he and why the Church have not engaged themselves in "advertising untried methods of relief," has met with a reply characteristic of Dr. Benson's dispassionate common sense. The Archbishop realises that there is a real danger in experimenting in new paths of charity and diverting support from old and tried institutions, hospitals, and the like. On this subject the Prelate writes, with somewhat positive assertion: "The net results," he says, "of such 'new developments' for charitable purposes has hitherto been 'simple subtraction' from support given to 'old and approved agencies.'" Surely it is wiser to strengthen the hands of existing efforts than to weaken all by too much scattered relief.

---

THE ultimate reason of punishment is prevention. The worse the crime, the severer the punishment. Hence the death of the murderer, as the supremest penalty, has been demanded by the laws of all peoples not only in past times, but these days of enlightenment also. Latterly the minds of many have been exercised as to whether this "life for a life" penalty is the most deterrent that can be inflicted, and not a few are inclined to think that if the crime of murder has diminished, the the result is due to civilisation and not to the fear of death. In three parts of America—Michigan, Rhode Island, and in Wisconsin—capital punishment has been abolished since 1853. It is remarkable that in these three States the crime of murder has been less frequent during the last decade, in ratio to the population, than in the other States, where there is no exemption of the penalty. Such a result certainly affords food for reflection.

IT appears that at the commencement of the cholera epidemic in Hamburg that the volunteers in the medical service were required, and it was understood at the time that medical students so volunteering were to receive 20 marks a day. Some misunderstanding was attached to this, but on the ground of this belief amongst the students, the Cholera Commission and Senate of Hamburg have decided to give the expected remuneration, and add to this their thanks to those who volunteered in the time of need.

---

ONE of the saddest growths of the present day is the tendency among the young to commit suicide. During the past year numerous instances of this have occurred, and doubtless all thoughtful people must have mentally searched for a cause Evidence seldom reveals the actual motive which cause these crimes. Nevertheless it is noteworthy that in more than one recorded instance the perpetrators of this unnatural crime have been habitual perusers of the most pernicious form light literature of the "penny dreadful" type. Taxation has been brought to bear on many of the necessities of this life; what a benefit would be conferred if Government would turn its attention to this profitless commodity, the "penny dreadful," and tax it off the earth.

---

THIS is very much the age of pensions. The enterprising will soon be at a loss for the subject to pension. The latest suggestion in this direction is that of pensioning old maids. Some provision, the philanthropist feels, should be made for the large number of women who, owing to the numerical inequality of the sexes, must perforce remain unmarried. The girls of a family have frequently no provision made for them if they miss the chance of marriage, and this is certainly very hard. They are not educated to gain a livelihood for themselves, and any efforts towards independence which they may make is not unusually resisted by the parent who eventually dooms them to poverty. We think ourselves that a moral tone requires to be established amongst parents as to their responsibilities in regard to their girls, and that any "pensioning" would but tend to lessen the parental sense of duty, and the unmarried women would be little better off in the end.

---

A CONTEMPORARY has recently commenced a crusade against the pernicious custom of tight-lacing, and really gruesome are some of the stories brought to our notice. The subject is not a new one. Doubtless the habit has been carried on to the detriment of many, in spite of medical warnings, ridicule, and personal inconvenience. But we are inclined to hope this latest crusade will not have such a difficult battlefield as former ones have had to fight on. Modern times certainly are inclining to common sense, some independence of opinion and athletics, amongst the fair sex. One and all of these are strong opponents to tight-lacing. The spirit of emulation will not allow Mary to permit Jane to triumph over her on the tennis ground, if letting out a reef or two will prevent it, and for these and other reasons we are inclined to believe health and comfort will be attained. English women, with the thoroughness that characterises their nation, have certainly gone to greater lengths with the pernicious habit than French women, for example. These, it is true, have exhibited figures wonderful to behold for an hour or two during the day; but the claims of society over, their costumes would not hamper dames of Venus di Milo proportions. English Society requires its women to be uniformly dressed throughout the day, a custom which i very hard on those who adopt the small waist.

